# A Cell-Based High-Throughput Screen for Novel Chemical Inducers of Fetal Hemoglobin for Treatment of Hemoglobinopathies

**DOI:** 10.1371/journal.pone.0107006

**Published:** 2014-09-16

**Authors:** Kenneth R. Peterson, Flávia C. Costa, Halyna Fedosyuk, Renee Y. Neades, Allen M. Chazelle, Lesya Zelenchuk, Andrea H. Fonteles, Parmita Dalal, Anuradha Roy, Rathnam Chaguturu, Biaoru Li, Betty S. Pace

**Affiliations:** 1 Department of Biochemistry and Molecular Biology, University of Kansas Medical Center, Kansas City, Kansas, United States of America; 2 Department of Anatomy and Cell Biology, University of Kansas Medical Center, Kansas City, Kansas, United States of America; 3 High Throughput Screening Laboratory, University of Kansas, Lawrence, Kansas, United States of America; 4 Department of Pediatrics, Georgia Regents University, Augusta, Georgia, United States of America; 5 Department of Molecular and Cell Biology, Georgia Regents University, Augusta, Georgia, United States of America; Broad Institute of Harvard and MIT, United States of America

## Abstract

Decades of research have established that the most effective treatment for sickle cell disease (SCD) is increased fetal hemoglobin (HbF). Identification of a drug specific for inducing γ-globin expression in pediatric and adult patients, with minimal off-target effects, continues to be an elusive goal. One hurdle has been an assay amenable to a high-throughput screen (HTS) of chemicals that displays a robust γ-globin off-on switch to identify potential lead compounds. Assay systems developed in our labs to understand the mechanisms underlying the γ- to β-globin gene expression switch during development has allowed us to generate a cell-based assay that was adapted for a HTS of 121,035 compounds. Using chemical inducer of dimerization (CID)-dependent bone marrow cells (BMCs) derived from human γ-globin promoter-firefly luciferase β-globin promoter-Renilla luciferase β-globin yeast artificial chromosome (γ-luc β-luc β-YAC) transgenic mice, we were able to identify 232 lead chemical compounds that induced γ-globin 2-fold or higher, with minimal or no β-globin induction, minimal cytotoxicity and that did not directly influence the luciferase enzyme. Secondary assays in CID-dependent wild-type β-YAC BMCs and human primary erythroid progenitor cells confirmed the induction profiles of seven of the 232 hits that were cherry-picked for further analysis.

## Introduction

Sickle cell disease (SCD) is the most common monogenetic disease diagnosed in the United States, affecting approximately 1 of 400 African-American infants [1]. The high morbidity rate of SCD patients is related to vascular complications that include multiple chronic organ damage affecting the brain, heart, lungs, kidneys, liver, eyes, skin, and skeleton. Vaso-occlusive crises result in acute and chronic severe pain, as well as acute chest syndrome, splenic sequestration, hemolytic anemia, stroke, acute and chronic multi-system organ damage, and shortened life expectancy [2], [3]. Understanding the molecular mechanisms underlying the human γ- to β-globin gene switch has long been recognized as important in the treatment of SCD, since a wealth of evidence has demonstrated that increased fetal hemoglobin (HbF) significantly ameliorates the clinical complications associated with this disease Individuals with defective adult β-globin genes, as is the case for SCD or β-thalassemia, are more-or-less phenotypically normal if they carry compensatory mutations that result in hereditary persistence of fetal hemoglobin (HPFH). Thus, a logical clinical goal for treatment of the β-hemoglobinopathies is to up-regulate γ-globin synthesis pharmacologically.

An increase in HbF parameters (% HbF and % F cells) prevents sickling *in vivo*. Hydroxyurea (HU), a ribonucleotide reductase inhibitor that arrests DNA synthesis, was shown to increase HbF production and improve the clinical symptoms of SCD [4], [5]. HU is currently the only FDA-approved drug to treat SCD, but the side effects and safety issues associated with long-term use are unknown [6]. Fetal hemoglobin synthesis can be stimulated by several other pharmacologic compounds including 5-azacytidine, AraC, butyrate and other short chain fatty acids [7], [8], [9], [10], [11], [12], [13], [14]. However, therapies specific for SCD, that do not affect cell physiology globally, remain elusive.

High-throughput (HTS) technology has been used successfully in the past 20 years in the pharmaceutical industry for lead drug discovery [15], [16]. Development of the technology has been propelled by advancements in combinatorial chemistry, human genome sequencing, and automation of drug screens. It is an enabling technology and the number of researchers that have applied this powerful tool in biomedical research has increased. Successful examples include discoveries of novel inhibitors of protein arginine methyltransferase [17], metalloform-selective methionine aminopeptidase [18], and cystic fibrosis transmembrane conductance regulator [19].

Based on our previous data using wild-type CID-dependent β-YAC BMCs, we generated dual-luciferase β-YAC (dual-luc β-YAC) BMCs containing γ-globin promoter-firefly luciferase (γ-luc) and β-globin promoter-Renilla luciferase (β-luc) fusions from γ-luc β-luc β-YAC transgenic mice. The γ-luc gene fusion was not active unless a γ-globin inducing compound was applied, a true “off-on” switch similar to our wild-type CID-dependent β-YAC BMCs, whereas the β-luc gene fusion was active. These cells were adapted to a HTS platform to allow rapid identification of chemical scaffolds that produced firefly luciferase-positive cells (γ-globin), but did not further induce Renilla luciferase (β-globin). Out of 121,035 compounds tested, 233 lead compounds that induce γ-globin gene expression were identified in this cell-based HTS system.

## Materials and Methods

Detailed Materials and Methods may be found in the accompanying Supplemental Materials and Methods file (File S1).

### Generation of the dual-luc β-YAC construct, production of transgenic mice and derivation of CID-dependent BMCs

**Figure 1:**
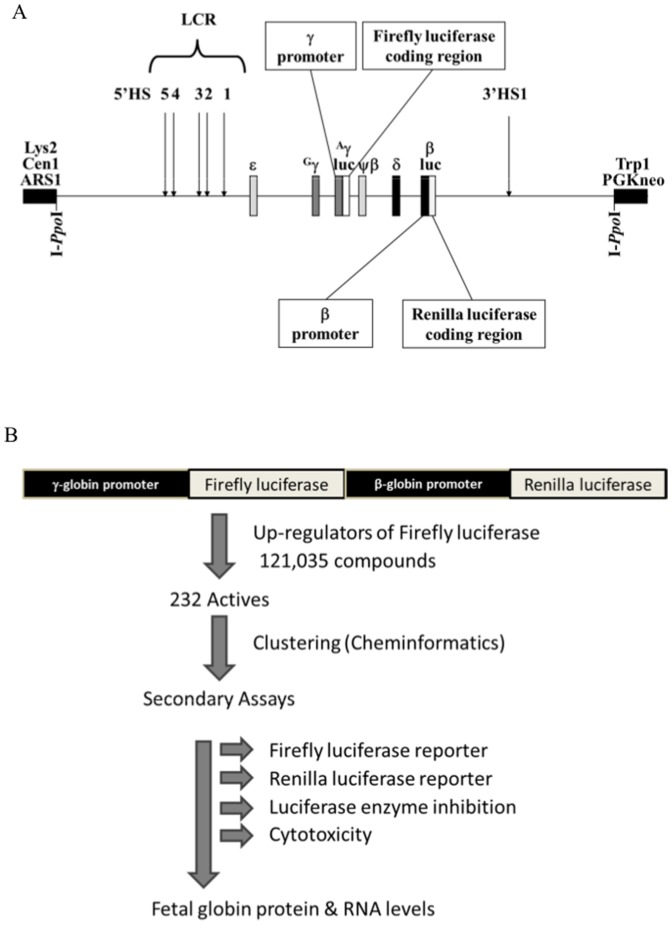
Schematic diagrams of the β-YAC construct and HTS work flow. Panel A) γ-luc β-luc β-YAC construct used to generate transgenic mice and derivative CID-dependent murine BMCs. This β-YAC was assembled as described in Materials and Methods and was derived from the *Ppo*-155 β-YAC [22]. The β-YAC is indicated as a line with the β-like globin genes or β-like globin promoter-luciferase fusions shown as boxes with the names of the genes above them. More detailed information regarding the components of the two luc fusions are indicated above and below the β-YAC illustration. Boxes at the left and right ends are modified pYAC4 vector [42] sequences. The LCR 5′HSs, 3′HS1 and YAC/yeast gene components are indicated above the line. LYS2, yeast lysine synthesis gene; ARS1, autonomous replicating sequence (yeast origin of replication); CEN1, yeast centromere; TRP1, yeast tryptophan synthesis gene; PGKneo, mammalian G418-resistance cassette. Engineered restriction enzyme sites utilized YAC structural determinations are shown below the line. **Panel B) High-throughput screening work flow and secondary assays.** The process flow of the high-throughput screen for identification of active compounds up-regulating fetal γ-globin gene expression is shown. The assay utilized immortalized multi-potential cells derived from the bone marrow of transgenic mice stably expressing a dual luciferase construct with firefly luciferase under control of the fetal ^A^γ-globin promoter and Renilla luciferase under the control of the adult β-globin promoter. The screening parameters were optimized in 384-well format and the cells were characterized for their ability to respond to at least 10 known inducers of fetal globin including hydroxyurea, sodium butyrate, valproic acid, and valeric acid. The assay was used to screen 120,035 compounds from the KU compound collection; 232 of which were found to up regulate firefly luciferase. The actives were clustered into 12 structural groups and fresh compounds were repurchased from various vendors. Three cell-based secondary assays were performed using the freshly available compounds: 1) up-regulation of firefly luciferase, 2) activity of Renilla luciferase, and 3) general cytotoxicity. The active compounds were also tested for inhibition of purified luciferase in an optimized biochemical assay. Profiling of the compounds revealed that of the 232 compounds tested, at least 124 compounds selectively up-regulated firefly luciferase but did not up-regulate Renilla luciferase. The 124 compounds which selectively up-regulated firefly luciferase activity also did not inhibit purified luciferase enzyme activity and were largely non-toxic to bone marrow progenitor cells.

A firefly luciferase gene cassette was incorporated into the ^A^γ-globin gene (Figure 1A) of a 155 Kb β-YAC [20] and the resultant γ-luc β-YAC was modified by introduction of a Renilla luciferase gene cassette into the β-globin gene using homologous recombination in yeast [21] for both steps, essentially as described [22]. The resultant β-YAC was purified and microinjected into fertilized oocytes to produce transgenic mice [23]. CID-dependent BMCs were established from these animals and maintained as previously [24].

### Ethics Statement

The mouse work was carried out in strict accordance with the recommendations in the Guide for the Care and Use of Laboratory Animals of the National Institutes of Health. The protocol was approved by the Institutional Animal Care and Use Committee of the University of Kansas Medical Center (ACUP Number: 2012–2060). All efforts were made to minimize suffering.

### High-throughput screening assay

For HTS experiments, the dual luc β-YAC BMCs were seeded into 384-well plates (Greiner Bio-One, Monroe, NC) at a density of 10,000 cells/30 µl/well by Wellmate bulk dispenser (ThermoScientific Inc., Waltham, MA) in complete media containing the CID, CL-COB-II-293 (synthesized by the University of Kansas COBRE CCET Core C Synthesis Lab, commonly called AP20187). The CID-dependent dual luc β-YAC BMCs were responsive to known γ-globin inducers including valproic acid, sodium butyrate, valeric acid and hydroxyurea (data not shown). Sodium butyrate (2 mM) was selected as the positive control for screening, as a consistent 5–6 fold increase in firefly luciferase activity was obtained in four independent experiments with four different passage numbers. Since both the cell viability and luciferase induction was unaffected at DMSO concentrations below 0.5%, the compound libraries were screened at 10 µM concentration in 0.35% DMSO. Compounds were transferred acoustically using a Labcyte Echo 550 dispenser (Labcyte Inc., Sunnyvale, CA). Based on previous optimizations, 10,000 cells/well in IMDM containing CL-COB-II-293 (100 nM) were added to the wells of 384-well assay plates. The negative (n = 16, DMSO) and positive (n = 16, sodium butyrate, 2 mM) controls were added to the first two columns of each plate to assure uniformity across plates and screening batches. The cells were exposed to compounds for 24 hours at 37°C, 5% CO_2_ in a 95% humidified incubator. After 24 hours incubation, Steady Glo luciferase detection reagent (Promega, Madison, WI) was used for cell lysis and generation of a luminescent signal, proportional to γ-globin promoter driven firefly luciferase reporter expression. The luminescence intensities were read 30 minutes later on a Tecan Safire2 microplate reader (Tecan, Männedorf, Switzerland). The luminescence values were used for calculating fold-induction of luciferase over DMSO treated controls. The controls were used to calculate a Z′ factor value for each plate, a measure of screening assay quality.

#### Compound libraries

For this study, the optimized cell-based assay utilized the following six compound collections: 1) MicroSource Spectrum (2,000 compounds containing FDA approved drugs, bioactive, natural products, MicroSource Discovery Systems, Gaylordsville, CT, www.msdiscovery.com), 2) Prestwick Chemical Library (1,120 compounds, Prestwick Chemical, ILLKIRCH France), 3) The University of Kansas Center of Excellence in Chemical Methodologies & Library Development (KU-CMLD, 1,920 compounds with novel diverse scaffolds), 4) ChemBridge Library (43,736 drug-like diverse chemical structures, ChemBridge Corporation, San Diego, CA, www.chembridge.com), 5) ChemDiv Library (56,232 compounds, diversity set from ChemDiv Inc., San Diego, CA), and 6) Orthogonally Compressed Library [(OCL), collection of 16,000 compounds from The Lankenau Institute for Medical Research (LIMR), Chemical Genomics Center (LCGC) Inc., Wynnwood, PA).

#### Data analysis

The active compounds from the primary screen were cherry-picked from mother plates and retested for firefly luciferase induction in an eight concentration dose-response assay (two fold dilution starting at 30 µM). The fold-induction values from dose-response curves were used for preliminary scaffold analysis and clustering using the Tripos Selector program and analyzed using the Jarvis Patrick routine, using default parameters [The Tripos Associates (Cetara), St. Louis, MO]. From each preliminary cluster, the largest conserved substructure present in at least half of the cluster members was identified.

#### Secondary screening

Some of the primary screen active compounds were repurchased and tested for activity in cell-based assays using the primary screening reporter cells in a ten concentration dose-response. Both firefly luciferase induction (γ-globin promoter activation using the One-Glo luciferase detection system; Promega, Madison, WI), and Renilla luciferase induction (β-globin induction the using Renilla-Glo Luciferase detection system; Promega, Madison, WI), were measured for specificity of compound activity. The cytotoxicity of the compounds was measured after 24 hours in the same cells using Cell-Titer Glo reagent (Promega, Madison, WI). The compounds were also tested in a biochemical screen for any inhibition to purified firefly luciferase enzyme (15 nM).

### Verification assays for γ-globin transcription and HbF synthesis in murine CID-dependent wild-type β-YAC BMCs or human CD34^+^ primary progenitor cells

Human erythroid progenitors were generated *in vitro* from adult CD34^+^ stem cells (STEMCELL Technologies, Inc., Vancouver, Canada) using a 2-stage culture system that achieves terminal erythroid differentiation [25]. Standard, but variant, methods for quantitative reverse transcription-PCR (qRT-PCR) and flow-activated cell sorting (FACS) were employed for the two cell types as detailed in File S1. ELISA was used to measure HbF in CID-dependent wild-type β-YAC BMCs as described in the supplement.

## Results

### Cell-based assay system characteristics

We developed CID-dependent ^A^γ-luc β-luc ββ-YAC BMCs from our transgenic mice as a powerful tool for screening activators of γ-globin [26]. This cell-based assay has a strong γ-globin gene expression off-on switch, a characteristic which is lacking in existing erythroid cell lines. A chimeric growth switch consisting of the thrombopoietin receptor (mpl) signaling domain fused to a FKBP12 ligand-binding domain is activated on addition of a CID. The CID, CL-COB-II-293 (AP20187), enforces dimerization by binding two FKBP12 ligand-binding domains on two neighboring molecules with 1∶2 stoichiometry. Dimerization causes signaling from the mpl receptor sequences. The resultant multi-potential transgenic BMCs express exclusively human β-globin from the wild-type β-YAC transgene [26]. γ-globin synthesis is not detected in wild-type β-YAC BMCs, but expression can be reactivated in the presence of 5-azacytidine (5-Aza), butyric acid and other fatty acids, hydroxyurea, or hemin.

The 150 Kb dual luc β-YAC was synthesized as described in Materials and Methods; a schematic diagram is shown in Figure 1A. Mouse L cells, a non-erythroid control, lipofected with the dual luc β-YAC, constitutively expressed γ-luc and β-luc, similar to wild-type β-YAC L cell lines and induction with hemin/HMBA was not observed (data not shown) [27], [28]. MEL585 cells or GM979 cells similarly transfected trended towards establishing proper luciferase expression patterns appropriate to each cell line with extended time in culture, but displayed mixed responsiveness to terminal differentiation agents or inducers, similar to cell lines produced with the wild-type β-YAC (data not shown). However, both firefly and Renilla luciferase activities steadily declined in cell pools or clones, whether induced or not, after 28 weeks of culture.

Transgenic mice were also produced with this dual luc β-YAC. The hematopoietic tissues of staged conceptuses were assayed for γ-firefly luciferase and β-Renilla luciferase. Generally, the two gene fusions showed correct developmental regulation, with γ-luc predominating during primitive erythropoiesis in the yolk sac and β-luc during the later stages of definitive erythropoiesis in the fetal liver and in the adult bone marrow (data not shown). CID-dependent dual luc β-YAC BMCs displayed a β-like globin gene expression pattern that was identical to previously established CID-dependent wild-type β-YAC BMCs, however γ-firefly-luc or β-Renilla-luc transcription/enzyme activity were the parameters measured, rather than native γ- or β-globin transcript/protein levels. In the absence of any HbF activating compounds, these cells were γ-luc^−^ β-luc^+^ (data not shown). Using this cell-based assay, a HTS and secondary verification assays were performed as outlined in Figure 1B.

### Primary HTS

**Figure 2:**
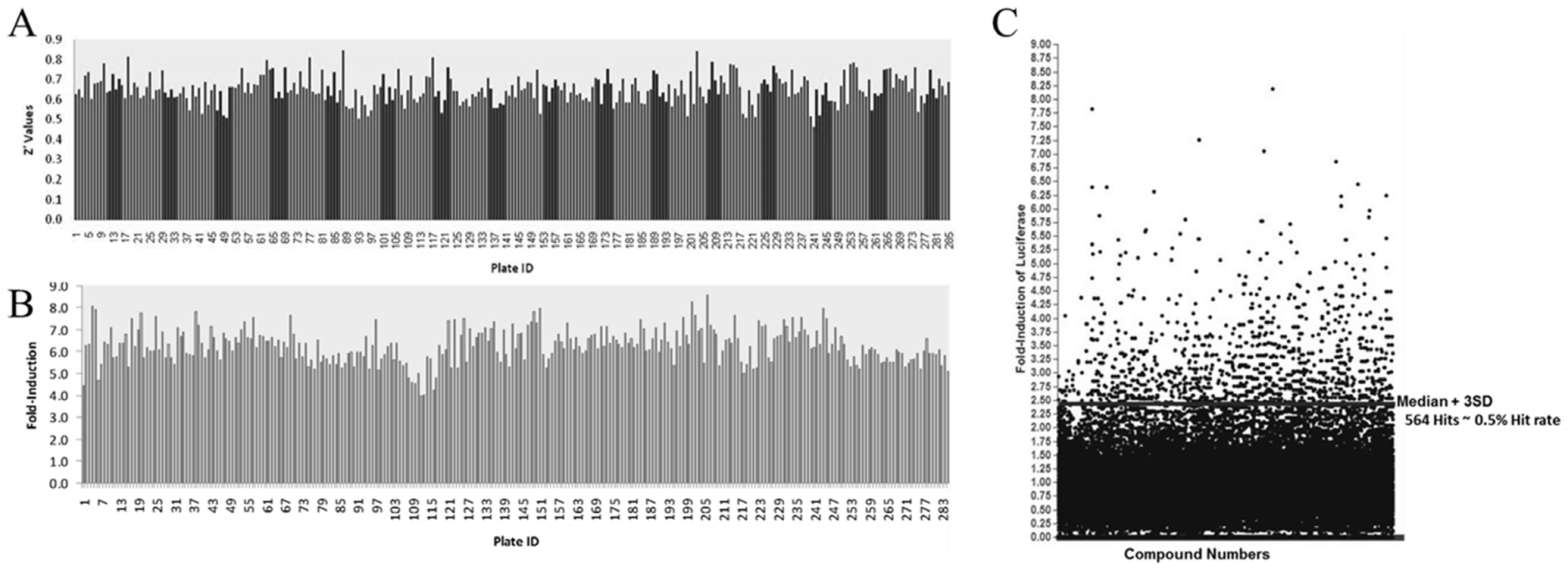
High-throughput screening for firefly luciferase inducers. **Panel A**) Distribution of Z′ scores. An average Z′ of 0.65±0.067 was obtained across all assay plates indicating suitability of the assay for high-throughput screening of 121,035 compounds. **Panel B**) Sodium butyrate-induced firefly luciferase expression. The treatment of cells with the positive control sodium butyrate resulted in an average increase in firefly luciferase expression by 6.3±0.77-fold across all 300 plates tested. **Panel C**) Scattergram of fold-induction of all 121,085 compounds tested using CID-dependent dual-luc β-YAC BMCs. 564 active compounds were identified that induced firefly luciferase greater than 3 standard deviations above the plate median.

Parameters for the HTS are described in Methods and outcomes are summarized in Tables 1 and 2. The screening assay was found to be robust with an acceptable dynamic range between the positive (sodium butyrate induction) and negative DMSO vehicle baseline controls across all the plates screened. The good separation of controls, as well as low variability around the means of the controls, resulted in an average Z′ factor value of 0.65±0.06 (Figure 2A) [29]. An average 6.3±0.7-fold induction of firefly luciferase was obtained with treatment of cells with 2 mM sodium butyrate (Figure 2B). The majority of compounds screened did not induce firefly luciferase. As shown in the scattergram of fold-induction of all 121,035 compounds screened (Figure 2C), only 564 compounds induced luciferase to greater than or equal to that of the plate median+3SD, resulting in a hit rate of 0.49%. A maximum fold-activation of >8-fold was obtained with some compounds in the primary screen. The actives from the primary screen were cherry-picked from mother plates and retested for firefly luciferase induction in an eight concentration dose-response (two fold dilution starting at 30 µM). Of 564 compounds, 328 compounds were dose-responsive with 83 compounds inducing luciferase to greater than 2.5 fold. The compounds were subjected to cheminformatics analysis to define the structural groups and the range of activities within each group (data not shown). At least 12 distinct clusters were identified using the Tripos Selector program. From each preliminary cluster, the largest conserved substructure present in at least half of the cluster members was identified. These data will used to guide efforts to progress these compounds forward as potential therapeutics and assist in choosing additional compound banks to screen.

**Table 1:** Primary screen.

Compound Source	No. of Compounds
Validation Library (Microsource, Prestwick and CMLD)	5,067
ChemBridge Library	43,736
ChemDiv Library	56,232
Orthogonally compressed library (LCGC)	16,000
Total No. of compounds	121,035

Actives: 232.

Overall hit rate: 0.19%.

**Table 2:** Reordered compounds.

Compound Source	No. of Compounds	Amount (mgs)
ChemBridge Library	171	2
ChemDiv Library	21	2
Orthogonally compressed library (LCGC)	40	2–5
Total No. of compounds	232	

100% DMSO: 50 or 100 mM.

### Secondary assays

**Figure 3:**
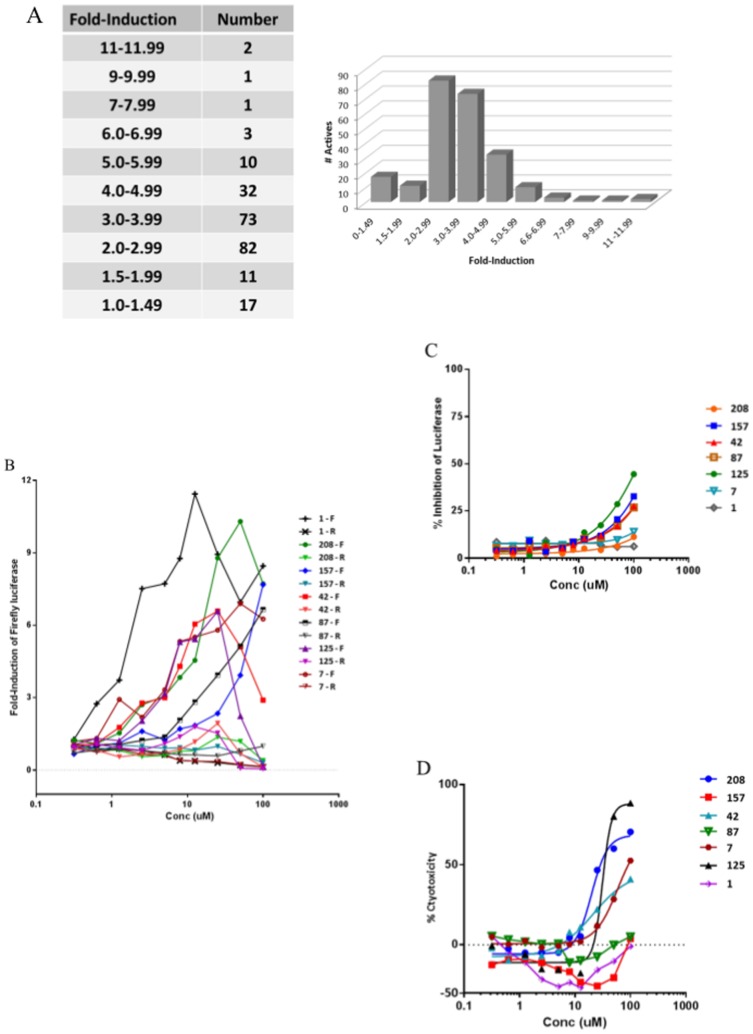
Reconfirmation and secondary assays of γ-globin inducer compounds. **Panel A) Reconfirmation of γ-luc inducibility by 232 actives from primary HTS.** Four secondary assays were employed including two reconfirmation assays for firefly luciferase induction and cytotoxicity, and two specificity assays for Renilla luciferase activity and luciferase enzyme modulators. All assays were a 10-point dose-response. A summary of firefly induction is shown in this figure. 211 of the 232 compounds had firefly induction of 2-fold or higher; a 90% reconfirmation rate. **Panels B-D) Performance of seven γ-firefly inducers in the initial four secondary assays – detailed 10-point dose-response data.** Comparison of compound activity from dose-response data is shown for firefly and Renilla luciferase activity (**Panel B**), purified firefly luciferase enzyme inhibition (**Panel C**), and cytotoxicity (**Panel D**). Assays were performed as described in Materials and Methods.

In order to establish that the firefly induction activity was not due to breakdown products of library compounds, some of the active compounds were repurchased as fresh powders (>95% purity). Of the 121,035 compounds screened, 232 actives that met the criteria outlined above, were identified and repurchased for further testing and validation. The number of actives within the 232 hit set identified in the primary screen of the CID-dependent dual luc BMCs that had γ-firefly luciferase induction 2-fold or higher was 211, thus 90% of the originally identified actives were reconfirmed (Figure 3A). The activity of the repurchased compounds was tested in a 10 concentration dose-response in the following 3 cell-based assays using the same CID-dependent dual luc BMCs used in the primary screen: 1) γ-firefly luciferase induction assay, 2) β-Renilla luciferase induction, and 3) cytotoxicity assays (Figure 1B). In addition to the three cell-based assays, a biochemical screen was set up using purified firefly luciferase to identify compounds that directly inhibit luciferase enzyme activity. Luciferase enzyme binders/modulators have been implicated in stabilizing luciferase RNA and hence appear as false positives in luciferase-based screens [30]. Based on these four secondary assays, at least 124 compounds were found to specifically induce firefly luciferase between 2- to 11-fold (Figure 3A), did not induce β-globin promoter-Renilla luciferase, and were not cytotoxic to the dual reporter-expressing BMCs (data not shown). None of the 124 compounds inhibited purified firefly luciferase.

Some of these compounds were tested in physiologically relevant non-reporter assay systems [31]. Seven cherry-picked repurchased compounds showed fold-induction of firefly luciferase (Figure 3B). The corresponding induction of Renilla luciferase activity is also shown in Figure 3B. These compounds were also tested for their ability to inhibit firefly luciferase enzyme in a biochemical assay (Figure 3C). The cytotoxicity of these compounds in CID-dependent dual luc β-YAC BMCs is shown in Figure 3D. All compounds were found to specifically up-regulate firefly luciferase with no significant induction of the Renilla reporter. While most of the compounds were not cytotoxic, a few compounds were found to affect cell viability at higher concentrations. Compounds #1 and #157 appear to be less cytotoxic than the control (Figure 3D); however in subsequent assays described below these compounds did not have a stimulatory effect on cell proliferation. Thus, the increased γ-globin signal was not a consequence of increased cell number.

Most of the firefly luciferase inducers did not significantly inhibit the luciferase enzyme (IC50>100 µM). Table 3 shows the EC50 values for the molar concentrations of these compounds resulting in 50% stimulation of firefly luciferase expression and their corresponding IC50 values for the molar concentrations resulting in 50% loss of cell viability. The therapeutic index for each compound was calculated from the ratio of IC50 for cytotoxicity to EC50 for luciferase stimulation. A larger window indicates significant separation of stimulatory activity of the compound from its cytotoxic effects. As shown in Table 3, the therapeutic windows were found to be significant for a majority of the compounds tested. Compound 1 with EC50 ∼1.4 µM and no detectable cytotoxicity exhibited the most favorable profile.

**Table 3:** EC50, IC50 and therapeutic index for seven cherry-picked compounds.

	Firefly Luciferase		Cytotoxicity	
Compound ID	EC50 (µM)	Fold-induction	IC50 (µM)	Therapeutic Index
1	1.4	8.6	>100	>100
7	5.9	6.5	87.6	15
42	9.8	5.2	>100	>100
87	49.3	9.6	>100	>100
125	5.7	7.0	35.0	6
157	30.0	9.5	>100	>100
208	14.3	10.1	24.3	2

>100: Less than 10% or no cytotoxicity up to 100 µM tested.

### Fetal hemoglobin activation in CID-dependent wild-type β-YAC BMCs

The HTS and initial validation of hits measured luciferase activity from fusions to the γ- and β-globin gene promoters. Thus, an important first secondary assay was to demonstrate that our actives effectively induced native γ-globin mRNA and protein, as well as the formation of HbF. For this purpose we utilized CID-dependent BMCs derived from wild-type β-YAC transgenic mice [26]. Criteria for cherry-picking compounds for testing in secondary assays included those with the highest fold induction, coupled with lowest toxicity. These are the top seven compounds listed in the table from Figure 3A. Compound #1 was dropped from further consideration because cell survival proved to be lower than expected based on the HTS results.

**Figure 4:**
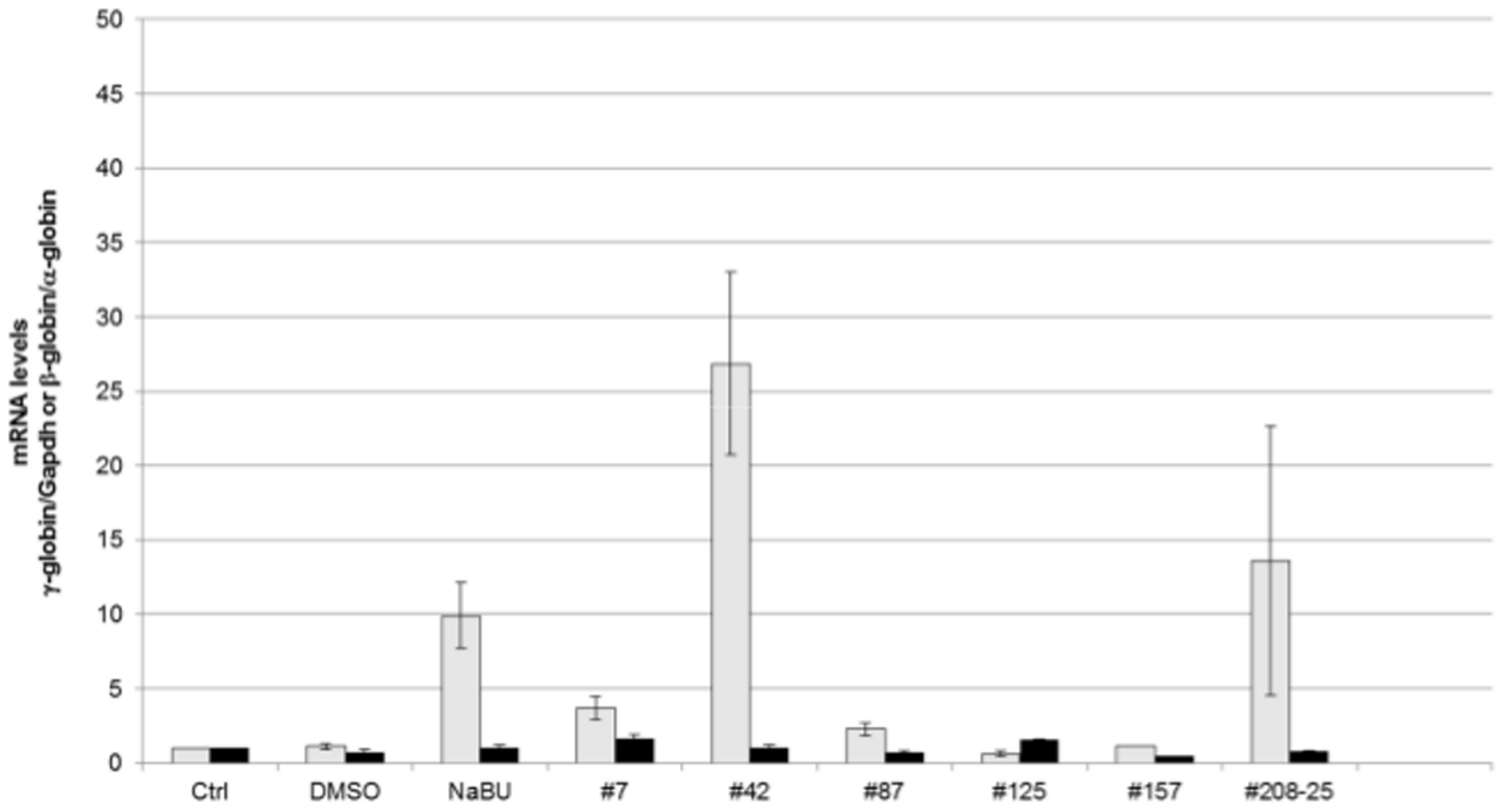
γ-globin and β-globin mRNA levels in compound-treated CID-dependent wild-type β-YAC mouse bone marrow cells. qRT-PCR was performed as described in Materials and Methods. Fold change in mRNA level is shown on the y-axis; sample names are shown on the x-axis. Ctrl, untreated cells; DMSO, cells treated with DMSO only; NaB, 2 mM sodium butyrate; #7, 50 µM; #42, 15 µM, #87; 100 µM; #125, 30 µM; #157, 100 µM; #208-25, 25 µM. Gray bars, γ-globin mRNA expression; black bars, β-globin mRNA expression. Data shown are the results of two-three separate experiments, with each sample duplicated within an experiment. P≤0.01 for NaB and compound # 42; P≤0.05 for compounds #42 and #208.

To confirm the effectiveness of these compounds, human γ- and β-globin gene expression was measured by qPCR in RNA samples from wild-type β-YAC BMCs treated with the 6 remaining compounds (Figure 4). γ-globin expression increased 26.9-fold with compound #42 and 13.6-fold with compound #208. Compounds #7 and #87 also induced γ-globin expression (3.7- and 2.3-fold, respectively). Sodium butyrate (NaB) and DMSO treatment was performed in parallel as a positive control and solvent control, respectively; 9.9-fold induction of γ-globin expression was observed with NaB treatment. Two of the compounds did not increase γ-globin transcription (#125 and #157). No induction of adult β-globin was observed with any of the 7 compounds tested.

**Figure 5:**
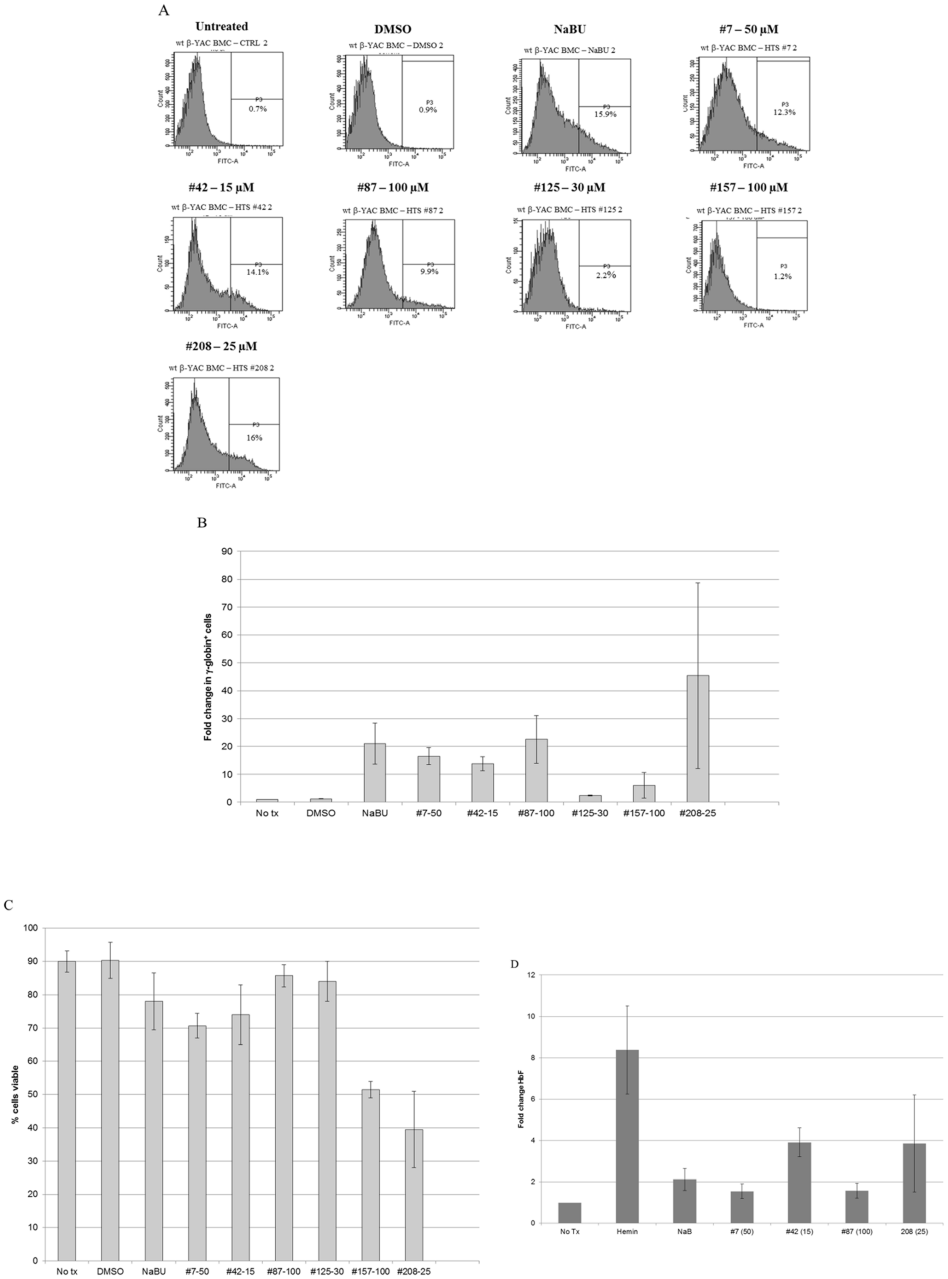
Induction of HbF by lead compounds in CID-dependent mouse BMCs. **Panel A) FACS analysis of HbF levels in compound-treated CID-dependent wild-type β-YAC mouse bone marrow cells.** The protocol was carried out as described in Materials and Methods using anti-human HbF FITC-conjugated antibody. Samples are labeled as described in the legend to Figure 4, but compound concentration follows the compound number. Representative data from one experiment is shown here, but the experiment was replicated two to three times for each sample with similar results (summarized in Panels B and C). **Panels B-C) Summary of FACS analysis of HbF levels in compound-treated CID-dependent wild-type β-YAC mouse bone marrow cells. Panel B)** fold change in γ-globin (FITC)^+^ cells. **Panel C)** percent viable cells. Bars show mean and standard error of the mean in control and compound-treated cells for each panel. **Panel D) HbF induction measured by ELISA in compound-treated CID-dependent wild-type β-YAC mouse bone marrow cells.** The assay was carried out as described in Materials and Methods. Fold induction of HbF is shown on the y-axis; cell treatment and concentration, where applicable, are shown on the x-axis. Data represent the mean and standard error of the mean from four experiments with duplicate samples within each experiment.

γ-globin (HbF)-expressing F cells were measured in parallel to confirm the induction of γ-globin expression at the protein level. Compound #208 showed 16% F cells and compound #42 had 14.1% F cells, compared to 15.9% F cells with NaB treatment and no change in F cells in DMSO only-treated and untreated samples (Figure 5A). These results are consistent with the induction of γ-globin observed at mRNA levels. Interestingly, compounds #7 and #87 showed higher induction of γ-globin at the protein level than mRNA, with 12.3% and 9.9% F cells (Figure 5A). The change in number of cells expressing γ-globin ranged from 14-46-fold (compounds #125 and #157 not included) compared to untreated cells, and in general, mirrored the % F cells measured (Figure 5B). Compound #42 was an exception with fewer cells expressing γ-globin compared to the number of F cells, whereas the opposite was true for compound # 87 and especially for compound #208. For compound # 208 this outcome might have been related to lower viability of cells following treatment (Figure 5C).

Based on the RT-qPCR and flow cytometry data for the six compounds assessed, #125 and #157 were dropped from further analyses based on lack of γ-globin transcript induction. Change in HbF was measured by ELISA following treatment with the remaining compounds in the wild-type β-YAC BMCs, as described in Materials and Methods. The pattern of expression of HbF by ELISA was similar to the mRNA expression results (Figure 5D). Treatment with compound #42 produced the greatest increase in HbF with the least variability, followed by compound #208 (with much greater variability), and then compound #7. The smallest response was seen with compound #87. The variation in response to #208 may be related to the much larger decrease in viability we observed with this compound (40%; Figure 5C)

### Fetal hemoglobin activation in human primary erythroid progenitor cells

**Figure 6:**
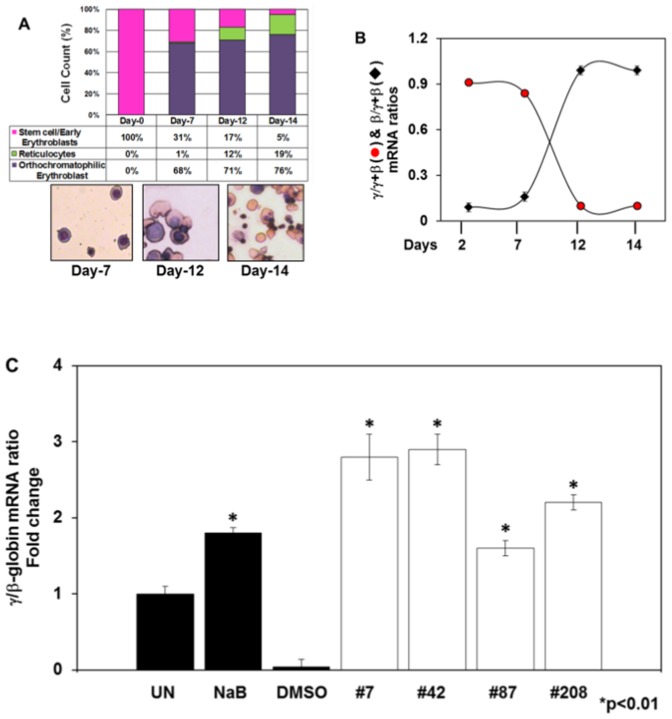
γ-globin expression is induced in compound-treated human primary erythroid progenitors. Human erythroid progenitors were generated from adult CD34^+^ cells in liquid culture as described in Materials and Method). Cells were analyzed for morphology, globin gene expression and HbF induction after treatment with the lead compounds. **Panel A**) Erythroid cells were harvested on the days indicated for cell morphology determination by Geimsa staining. The percentage of different erythroid progenitors at each stage is shown as a function of days in culture. At least 500 cells were counted by light microscopy from duplicate slides. Representative cell morphology is shown in the images at 40× magnification. **Panel B**) qRT-PCR was performed as described in Materials and Methods. The levels of γ- and β-globin expression were normalized to GAPH; expression of each gene is shown as a fraction of the total globin (γ+β). Note the γ-to β-globin switch around day 10. **Panel C**) Fold change in γ-globin/β-globin mRNA ratio after normalization to GAPDH is shown on the y-axis. UN, untreated cells; DMSO, cells treated with DMSO only; NaB, 2 mM sodium butyrate; lead compounds: #7, 50 µM; #42, 15 µM, #87; 100 µM; #208, 25 µM. Data shown are the mean and standard error of the mean of three independent samples.

Our last set of studies was aimed at demonstrating the ability of the lead compounds to induce HbF expression in human erythroid progenitors generated in liquid cultures. CD34+ cells were cultured in a 2-stage system as described in Materials and Methods. Initial studies were performed to monitor terminal erythroid maturation and the γ- to β-globin switch in this system. Cells were harvested every 2–3 days and morphology was examined by Giemsa staining. We observed a steady progression of erythropoiesis with 19% reticulocytes and mature red blood cells by day 14 (Figure 6A). We next performed qRT-PCR to follow the γ- to β-globin switch, which was observed around day 10 in culture (Figure 6B). These data support the use of this system to test the ability of lead compounds to activate human γ-globin. The various compounds were added on day 8 for 48 hours and cell viability, measured by trypan blue exclusion, remained greater than 97% throughout the culture period. Using qRT-PCR, the γ-globin to β-globin (γ/β) ratio was increased 1.8-fold for the positive control sodium butyrate when compared to DMSO (Figure 6C). In contrast, the γ/β ratio for lead compounds #7, #42, #87 and #208 was increased 2.8-, 2.9-, 1.6- and 2.2-fold, respectively.

**Figure 7:**
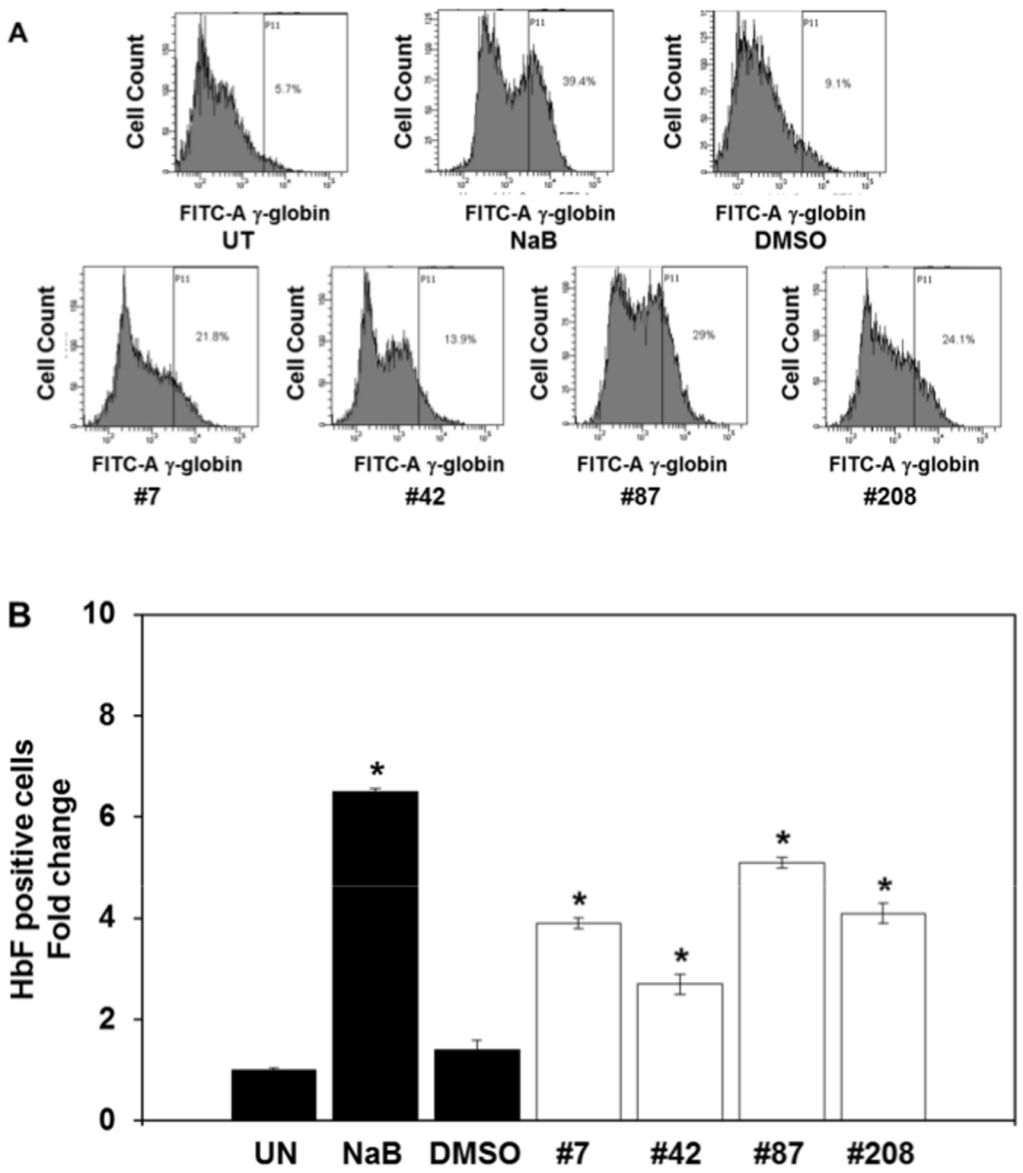
The lead compounds induce HbF expression in human primary erythroid cells. FACS analysis was performed with erythroid progenitors treated for 48 hours with each of the four lead chemical compounds. The protocol was carried out as described in Materials and Methods using anti-human HbF FITC-conjugated antibody. **Panel A**) Representative FACS tracings are shown; experiments were replicated three times for each compound with similar results. **Panel B**) Based on the FACS data the % HbF-positive cells (FITC-A) was calculated for the different treatment conditions. Shown in the graph is the fold change in HbF-positive cells. Data are shown as the mean and standard error of the mean in control and compound-treated cells for each sample. *; P<0.01.

FACS analysis was conducted to determine if the lead compounds induce HbF synthesis in erythroid progenitors by measuring the % HbF-positive cells (Figure 7A). We observed a 7-fold increase in % HbF-positive cells after sodium butyrate treatment compared to a 2.7-5.1-fold increase mediated by the lead compounds (Figure 7B). DMSO alone did not change the levels of HbF-positive cells significantly. These data demonstrate the ability of the lead compounds #7, #42, #87 and #208 to act as HbF-inducers in human primary erythroid cells.

## Discussion

Proteins with potential therapeutic value must meet the criteria implied or predicted for druggable targets based on evolutionary relationships, 3D-structural properties, features derived from amino-acid sequence or properties of ligands known to bind the protein. Proteins with narrow specificity for regulating γ-globin gene expression constitute potential ideal druggable targets, but none have been identified. Alternate approaches utilized molecular modeling to identify new therapeutic candidates based on compounds previously shown to induce HbF, such as short-chain fatty acids and derivatives [32], a chemical genetic strategy in primary cells for HDAC1 and 2 inhibitors [33], an optical assay in which the color is proportional to the anti-sickling effect of the test compound [34] or a more naïve cell-based γ-globin expression reporter HTS assay [35]. This latter approach relied on phenotype (γ-globin induction, enhanced HbF expression) to identify useful agents, rather than searching for compounds or ligands that directly affect a target protein. The lack of fetal γ-globin-specific regulatory proteins that meet druggability criteria has made this approach the method of choice for identification of potential new therapeutic compounds to treat β-hemoglobinopathies. Research on γ-globin gene regulation may result in the discovery of fetal-specific activators or repressors that may be druggable. In the meantime, phenotypic selection for HbF chemical inducers using assays amenable to HTS of large compound libraries will allow exhaustive assessment of all available compound and drug libraries.

The reporter assay in the cell-based HTS mentioned above was not robust [34], but the cell-based system we developed provides a tightly-regulated γ-globin expression “off-on” switch with a readout that was adapted for HTS. Identification of pharmacologic agents capable of reactivating γ-globin gene expression has been complicated by the lack of suitable cell lines. Essentially, cell lines were not available that displayed a suitable “off-on” switch for γ-globin gene expression, including mouse human globin transgenic MEL cells, HFE-MEL switching hybrids, and human K562 or HEL cells. We rationalized that a better approach would be to use primary progenitors derived from β-YAC transgenic mouse bone marrow using a novel method to immortalize these cells [24], [26], [36]. A CID was used to specifically and reversibly control the growth of primary, multi-potential hematopoietic cell populations *in vitro* and establish cell culture. In CID-dependent wild-type β-YAC BMCs, β-globin is expressed, but γ-globin expression is not detectable. However, expression is inducible using a variety of drugs known to increase HbF. Endogenous mouse β^maj^-globin and α-globin are also synthesized [26]. Addition of erythropoietin (Epo) and stem cell factor (SCF) modestly enhance globin gene expression; a cocktail of Epo, granulocyte monoctye-colony stimulating factor (GM-CSF) and interleukin-3 (IL-3) does not. BMCs derived from -117 Greek HPFH β-YAC transgenic mice [37] recapitulated the HPFH phenotype. A γ-globin-specific synthetic Zn finger activator protein (gg1-VP64) [38], [39] induced γ-globin synthesis in wild-type β-YAC BMCs, as does enforced expression of other fetal-specific transcriptional activators including FKLF and FGIF [26]. Differentiation into terminal erythrocytes is not required, since globin gene expression patterns are similar in both our BMC and erythroid cell populations.

CID-dependent bone marrow cells were established from ^A^γ-luc β-luc β-YAC transgenic mice. These cells were utilized to develop and benchmark a robust HTS assay to identify compounds that 1) induced γ-globin gene expression, 2) did not induce β-globin gene expression, 3) were not cytotoxic and 4) did not directly affect luciferase enzymatic activity. The HTS surveyed 121,035 compounds from various libraries and yielded 232 hits that induced γ-luc 2- to 11-fold. Of these, 211 were reconfirmed for γ-luc induction, and 124 met the constraints of the four listed criteria following a 4-assay, 10 point dose-response secondary screen. Six of the top 124 actives were cherry-picked for further analysis and conformation in other cell-based systems.

Our first assessment was in wild-type β-YAC BMCs, where normal γ-globin transcript and protein levels could be measured in the multi-potential mouse BMC background, rather than luciferase activity. Using the optimum dose for each compound, four of the six compounds induced γ-globin transcription 2- to 26-fold and had increased F cells ranging from 9.9 to 16% in wild-type β-YAC BMCs. ELISA concurred with these data; an approximately 2- to 4-fold increase in HbF was observed; the same pattern of change seen in mRNA levels was reflected in protein levels.

Although different cell lines have been used extensively to screen chemical compound libraries for HbF inducers, these lines do not undergo hemoglobin switching. Thus, the output of these screens may yield false-positive hits that do not correlate with their ability to induce HbF in primary human erythroid progenitors. The dual luciferase reporter system established in primary mouse BMCs immortalized by the CID and used for the HTS described in this report more closely mimics human erythroid progenitors. Our studies in erythroid progenitors generated from adult human primary CD34^+^ stem cells confirmed the validity of using the CID-dependent β-YAC BMCs for the HTS. In addition, the data demonstrated that this system recapitulated erythropoiesis and the γ to β-globin switch in a manner similar to that previously published using another liquid culture system [40], [41]. The advantage of the human progenitor terminal differentiation protocol is that we produce mature erythrocytes and mimic the γ- to β-globin switch similar to normal erythropoiesis *in vivo*, thereby adding further credibility to our hit identification. The fact that HbF was induced by lead compounds #7, #42, #87 and #208 to levels greater than sodium butyrate suggests that these agents are candidates for further drug development using medicinal chemistry approaches to treat β-hemoglobinopathies.

## Supporting Information

File S1 Supplemental Materials and Methods.
(DOCX)
