# Supplementary material for: A Cell-Based High-Throughput Screen for Novel Chemical Inducers of Fetal Hemoglobin for Treatment of Hemoglobinopathies
Source: PLoS One. 2014 Sep 16;9(9):e107006. doi: 10.1371/journal.pone.0107006 (PMC4165891; doi:10.1371/journal.pone.0107006)
Supplement: File S1 — Supplemental Materials and Methods. (DOCX) [file pone.0107006.s001.docx]

SUPPLEMENTAL MATERIALS AND METHODS

*Generation of the dual-luc β-YAC construct, production of transgenic mice and derivation of CID-dependent BMCs*:

We incorporated, through yeast integrating plasmid (YIP)-mediated homologous recombination in yeast using the “pop-in”, “pop-out” method [1], a firefly luciferase gene cassette into the ^A^γ-globin gene (Figure 1A) of a 150 Kb β-YAC [2]. The pRS406 YIP vector (Stratagene, La Jolla, CA) contained the following fragments ligated between the *Hind*III and *Eco*RI restriction enzyme sites of pRS406 to product the ^A^γ-globin promoter-firefly luc fusion. They were, in order, a 1.4 Kb *Hind*III-*Nco*I 5’ ^A^γ-globin fragment containing the promoter (Genbank file, coordinates 38,085-39,482), a 2.7 Kb *Xho*I-*Sal*I luciferase cassette from pGL2 Basic (Promega, Madison, WI) and a 2.4 Kb *Eco*RI 3’ ^A^γ-globin gene containing a region just downstream of the end of the gene (Genbank coordinates 41,391-43,741). The plasmid was linearized at a unique *Nhe*I site in the 3’ ^A^γ-globin region (Genbank coordinate 43,203) prior to transformation of *Ppo*-155 Kb β-YAC-bearing yeast [3] for the homologous recombination steps. The ^A^γ-globin coding region was completely deleted and replaced by the luciferase gene.

The resultant γ-luc β-YAC was similarly modified to introduce a Renilla luciferase gene cassette into the β-globin gene (Figure 1A); the pRS406 vector was utilized. A 4.1 Kb *Hpa*I-*Xba*I β-globin gene fragment (Genbank coordinates 61,373-65,474) was inserted into the vector and a 1 Kb *Nhe*I-*Xba*I Renilla luciferase fragment from pRL-null (Promega) was ligated into the resultant vector to replace a *Nco*I-*Bam*HI β-globin sequence encompassing exon 1 through most of exon 2 (Genbank coordinates 62,238-62,686), linking the luc cassette to the β-globin promoter. The plasmid was linearized at a unique *Eco*RI site in the 3’ region downstream of the β-globin coding region (Genbank coordinate 63,583) prior to yeast transformation. The β-YAC containing the human β-globin locus with the ^A^γ-globin promoter-luc and β-globin promoter-luc fusions was purified and microinjected into fertilized oocytes to produce transgenic mice [4] and CID-dependent BMCs were established as previously described [5]. This work was carried out in strict accordance with the recommendations in the Guide for the Care and Use of Laboratory Animals of the National Institutes of Health. The protocol was approved by the Institutional Animal Care and Use Committee of the University of Kansas Medical Center (ACUP Number: 2012-2060). All efforts were made to minimize suffering.

*Cell growth and maintenance*:

The dual luc β-YAC and wild-type β-YAC BMCs were maintained in Iscove’s Modified Dulbecco’s medium (IMDM) containing L-Glutamine (Invitrogen, Carlsbad, CA) supplemented with 10% heat-inactivated fetal bovine serum, sodium pyruvate, non-essential amino acids and the CID, CL-COB-II-293 (synthesized by the University of Kansas COBRE CCET Core C Synthesis Lab, commonly called AP20187). The cells were passaged at 3 X 10^5^ cells/ml two to three times a week in the presence of CL-COB-II-293, incubated at 37^0^C, 5% CO_2_ in a 95% humidified incubator. For HTS experiments, the dual luc β-YAC BMCs were seeded into 384-well plates (Greiner Bio-One, Monroe, NC) at a density of 10,000 cells/30 μl/well by Wellmate bulk dispenser (ThermoScientific Inc., Waltham, MA) in complete media containing CL-COB-II-293. For validation assays, wild-type β-YAC BMCs were seeded at 50,000 cells per well and non-transgenic BMCs were seeded at 100,000 cells per well. Cells were treated as described below.

*Verification assays*.

*a) γ-globin transcription and HbF synthesis in murine CID-dependent wild-type β-YAC BMCs*.

Quantitative reverse transcription-PCR (qRT-PCR). In each experiment, treated and non-treated samples were analyzed in duplicate, 48 hours post-treatment, using SYBR Green dye in a CFX96 instrument (Bio-Rad, Hercules, CA). Expression of γ- and β-globin was calculated using relative quantification, as previously described [6], [7], using samples from β-YAC transgenic mice as controls. PCR primer sequences utilized for expression studies were: Hu-γ1, 5’-GACCGTTTTGGCAATCCATTTC-3’; Hu-γ2, 5’-GTATTGCTTGCAGAATAAAGCC-3’; β-globin FWD, 5’-GAGAAGTCTGCCGTTACTGCC-3’; β-globin REV, 5’-CCGAGCACTTTCTTGCCATGA-3’; Mo-Gapdh FWD, 5’-AGGTTGTCTCCTGCGACTTCA-3’; Mo-Gapdh REV, 5’-CCAGGAAATGAGCTTGACAAAG-3’; Mo-α-globin FWD, 5’-GATTCTGACAGACTCAGGAAGAAAC-3’; Mo-α-globin REV, 5’-CCTTTCCAGGGCTTCAGCTCCATAT-3’. Murine Gapdh and α-globin gene expression were used as internal controls for the human β-like globin gene expression. Mean and standard error of the mean were calculated. The Student’s t-test was used to determine statistical significance at P < 0.05 and P < 0.01.

Flow-activated cell sorting (FACS). Detection of HbF in CID-dependent wild-type β-YAC BMCs was performed by flow cytometric analysis. Briefly, 3 x 10^6^ cells were cultured for 48 hours with or without treatment. Cells were washed in PBS and fixed in 1 ml 4% fresh paraformaldehyde (Sigma Aldrich, Saint Louis, MO). The cells were centrifuged, the supernatant discarded, and the pellets were resuspended in 1 ml ice cold acetone:methanol (4:1) for 1 minute. Cells were washed twice in ice-cold PBS/0.1% BSA and resuspended in 100 μl PBS/0.1% BSA/0.01% Tween 20 (PBT). One μg sheep anti-human HbF FITC-conjugated antibody (A80-136F, Bethyl Laboratories, Montgomery, TX) in 100 μl PBT was added to the cell suspension and incubated for 40 minutes at room temperature. Cells were washed with 1 ml ice-cold PBS/0.1% BSA and the pellets were resuspended in 300 μl PBS/2.5% FBS for flow cytometry. Cells were analyzed using a BD LSRII Flow Cytometer (BD Biosciences, San Jose, CA) with a 530/30 nm (FITC/GFP) emission filter . Data from 30,000 events was acquired for analysis employing BD FACS Diva software.

ELISA. CID-dependent wild-type β-YAC BMCs (50,000/well) or non-transgenic BMCs (100,000 cells/well) were cultured in duplicate in 96-well flat-bottom microplates (BD Biosciences, San Jose, CA) with compounds at the indicated concentrations. Controls included cells that were not treated or were induced with 1 mM NaB or 20 μM hemin. After 2-day incubation, cells were transferred to a round-bottom microplate (BD Biosciences, San Jose, CA) and centrifuged at 200 x g. The supernatant was removed, 200 μl PBS was added, and the cells were centrifuged again. The supernatant was removed, 100 μl lysis buffer [50 mM Tris, pH 8.0, 0.14 M NaCl, 0.5% TritonX-100, 100 μg/ml PMSF (protease inhibitor cocktail, 04693124001, Roche, Indianapolis, IN)] was added, and the microplates were frozen at -80^o^C.

Griener high-binding ELISA plates (M4561-40EA, Sigma-Aldrich, St. Louis, MO) were coated with 100 μl of 7.5 μg/ml sheep anti-human HbF antibody (A80-136, Bethyl Laboratories, Montgomery, TX) in coating buffer (0.5 M carbonate/bicarbonate buffer, pH 9.6) and incubated overnight at 4^o^C. Wells were washed 5 times in wash buffer (50 mM Tris, pH 8.0, 0.14 M NaCl, 0.02% Tween-20). Wells were blocked with 300 μl block buffer (10% FBS in PBS, 0.02% Tween-20) for one hour at room temperature. Wells were washed 5 times in wash buffer. Microplates of induced cells were subjected to 3 freeze/thaw cycles. 75 μl of each lysate was added to wells of the prepared ELISA plates and incubated one hour at room temperature. The remaining lysate was used in a protein assay as described below. Wells were washed 5 times in wash buffer, then incubated with 100 μl mouse monoclonal anti-human HbF (10C-CR8115M1, Fitzgerald, Acton, MA) diluted 1:10,000 (to 0.854 μg/ml) in block buffer for one hour at room temperature. Wells were washed 5 times in wash buffer, and then incubated for one hour at room temperature with goat anti-mouse IgG-HRP (sc-2055, Santa Cruz Biotechnology, Santa Cruz, CA) diluted 1:5,000 (to 0.08 μg/ml) in block buffer. Wells were washed 5 times in wash buffer, and then developed by addition of 100 μl peroxide/substrate solution (34021, Thermo Scientific, Rockford, IL) for one hour at room temperature. Development was stopped with 100 μl 2 M sulfuric acid. The solution was transferred to a clear bottom black plate (Costar 3603, Corning, Corning, NY) and read at 450 nm in a microplate spectrophotometer (SpectraMax M5, Molecular Devices, Sunnyvale, CA). Protein concentration was measured in a 96-well assay plate by mixing 5 μl of each sample or control with 250 μl Quickstart Bradford Dye Reagent (500-0205, BioRad, Hercules, CA). Plates were incubated for 15 minutes before they were read at 595 nm in a microplate spectrophotometer.

Values for the ELISA were calculated as follows. HbF absorbance of CID-dependent wild-type β-YAC BMCs was adjusted for background by subtracting the average absorbance of multiple wells of non-transgenic BMCs. The ratio of the adjusted HbF absorbance to the total protein absorbance of each well was calculated. Fold induction of each sample was then calculated by dividing the HbF/total protein ratio of each treated well by the same ratio of un-treated well. Fold induction of four replicates was then averaged, standard error of the mean was calculated and these values were plotted.

*b) γ-globin transcription and HbF synthesis in human CD34^+^ primary progenitor cells*.

Human primary erythroid cell culture. Human erythroid progenitors were generated *in vitro* from adult CD34^+^ stem cells (STEMCELL Technologies, Inc., Vancouver, Canada) using a 2-stage culture system that achieves terminal erythroid differentiation [8]. Briefly, CD34^+^ stem cells (500,000) were grown in First medium consisting of IMDM containing human AB serum, Interleukin-3 (IL-3,10 ng/ml), stem cell factor (SCF, 10 ng/ml) and erythropoietin (EPO, 2 IU/ml). On day 7 the erythroblasts were placed in Second medium consisting of 2 IU/mL erythropoietin for the duration of culture. Cells were harvested every 2-3 days; qRT-PCR was employed to construct the globin switching curve; Geimsa staining was used to determine cell morphology; and cell counts were performed by light microscopy. Cell viability was monitored by trypan blue (0.4%) exclusion. On day 8, erythroid progenitors were treated with sodium butyrate (2 mM), and lead compounds #7 (5 μM), #42 (15 μM), #87 (25 μM) and #208 (25 μM) for 48 hours; DMSO was tested as the vehicle control. Cells were also harvested for FACS analysis.

Quantitative reverse transcription-PCR (qRT-PCR). The mRNA levels of γ-globin, β-globin, and glyceraldehyde-3-phosphate dehydrogenase (GAPDH) were measured as previously published [9], [10]. Total RNA was extracted from three independent cultures using TRIzol reagent (Invitrogen, Grand Island, NY) according to the manufacturer's instructions. The different cDNAs were prepared from total RNA (1 µg) using the Improm-II RT system (Promega, Madison, WI). The γ-globin, β-globin, and GAPDH mRNA levels were quantitated by Sybergreen qPCR using an iCycler iQ instrument (BioRad, Hercules, CA); standard curves were generated using a Topo7-based plasmid carrying the γ-globin, β-globin, or GAPDH cDNAs (Topo7-γ-globin, Topo7-β-globin, or Topo7-GAPDH, respectively).

Flow-activated cell sorting (FACS). After compound treatments, 500,000 erythroid progenitors were washed twice with phosphate-buffered saline, fixed in 4% paraformaldehyde and permeated with ice-cold acetone/methanol (4:1). Cells were incubated with anti-γ-globin FITC-conjugated antibody (Santa Cruz Biotechnology, Santa Cruz, CA) in PBT (PBS/01% BSA/0.1% Triton X-100) solution for 20 minutes. The labeled cells were analyzed using a Becton-Dickson LSR-II flow cytometer (BD Biosciences, San Jose, CA). All experiments were performed in triplicate.

SUPPLEMENT REFERENCES

1. Rothstein, R (1983) One-step gene disruption in yeast*.* Meth Enzymol 101: 202-210.
2. Gaensler KM, Burmeister M, Brownstein BH, Taillon-Miller P, Myers RM (1991) Physical mapping of yeast artificial chromosomes containing sequences from the human β-globin gene region. Genomics 10: 976-984.
3. Peterson KR, Navas PA, Li Q, Stamatoyannopoulos G (1998) LCR-dependent gene expression in β-globin YAC transgenics: detailed structural studies validate functional analysis even in the presence of fragmented YACs. Hum Mol Genet 7: 2079-2088.
4. Peterson KR, Clegg CH, Huxley C, Josephson, BM, Haugen HS, et al. (1993) Transgenic mice containing a 248-kb yeast artificial chromosome carrying the human β-globin locus display proper developmental control of human globin genes. Proc Natl Acad Sci USA 90: 7593-7597.
5. Blau CA, Peterson KR (2006) Establishment of cell lines that exhibit correct ontogenic stage-specific gene expression profiles from tissues of YAC transgenic mice using chemically induced growth signals. In: MacKenzie, A, editor. Methods in Molecular Biology, Vol. 349: YAC Protocols, 2nd ed. Totowa, NJ: Humana Press. pp. 163-173.
6. Livak KJ, Schmittgen TD (2001) Analysis of relative gene expression data using real-time quantitative PCR and the 2^-ΔΔCT^ method. Methods 25: 402-408.
7. Pfaffl MW (2001) A new mathematical model for relative quantification in real-time RT-PCR. Nucleic Acids Res 29: e45.
8. Hebiguchi M, Hirokawa M, Guo YM, Saito K, Wakui H, et al. (2008) Dynamics of human erythroblast enucleation. Int J Hematol 88: 498-507.
9. Sangerman J, Lee MS, Yao X, Oteng E, Hsiao CH, et al. (2006) Mechanism for fetal hemoglobin induction by histone deacetylase inhibitors involves γ-globin activation by CREB1 and ATF-2. Blood 108: 3590-3599.
10. Ramakrishnan V, Pace, BS (2011) Regulation of γ-globin gene expression involves signaling through the p38 MAPK/CREB pathway. Blood Cells Mol Dis 47: 12-22.
